# A partial pathway- and network-based transformation reveals the synergistic mechanism of JA and UA against cerebral ischemia-reperfusion injury

**DOI:** 10.1007/s13238-014-0098-0

**Published:** 2014-09-13

**Authors:** Shanshan Guo, Li Guo, Yanan Yu, Bing Li, Yingying Zhang, Haixia Li, Ping Wu, Jie Wang, Ye Yuan, Zhong Wang, Yongyan Wang

**Affiliations:** 1 Institute of Chinese Materia Medica, China Academy of Chinese Medical Sciences, Beijing, 100700 China; 2 Guang’anmen Hospital, China Academy of Chinese Medical Sciences, Beijing, 100700 China; 3 Institute of Basic Research in Clinical Medicine, China Academy of Chinese Medical Sciences, Beijing, 100700 China; 4 Institute of Information on Traditional Chinese Medicine, China Academy of Chinese Medical Sciences, Beijing, 100700 China

**Dear Editor,**

Ischemic stroke falls under the category of complex diseases, which do not obey the single-gene dominant or single-gene recessive Mendelian pattern of inheritance but rather arises from the combination of numerous genetic variants and environmental factors (Cho et al., 2012). Given this degree of complexity, a single compound could never be expected to manage or reverse the effects of a stroke. Recently there has been accumulating evidence suggesting that combination therapies could exert a significant effect on areas affected by a cerebral ischemic stroke. Combination therapies that simultaneously impact multiple targets have promise in the control of complex diseases by way of synergy (Li et al., 2011).

Jasminoidin (JA) and ursodeoxycholic acid (UA) are compounds extracted from Qingkailing injection, which was extensively used for treating acute ischemia stroke for decades in China (Cheng et al., 2012). There was synergistic effect observed with the combination of JA and UA with regard to the reduction of infarction volumes, a decrease in the neurological deficit score and the MRI results in ischemic rats (Liu et al., 2012). However, till now, little was known regarding the synergistic mechanism of JA and UA in the treatment of ischemia stroke until the implementation of an integrated network analysis of global gene expression profiling.

In the present study, we aim to investigate the synergistic mechanism of JA and UA against cerebral ischemia on a systemic level. The IPA system was applied to identify the differential networks and canonical pathways as well as to determine the overlap of these diverse biological functions using an integrated network analysis of the global gene expression profiling in the JA, UA, JU (JA + UA combination) treatment groups.

Firstly, the IPA results revealed 11 statistically significant networks between the JU and vehicle groups. The top 5 networks included 35, 35, 35, 34 and 35 nodes (genes; proteins) (Table.S1). Network 1 includes gene expression, cell cycle and cellular compromise (Fig. 1A); network 2 functions in DNA replication, recombination and repair, drug metabolism, and endocrine system development and function (Fig. 1B); network 3 involves endocrine system development and function, small molecule biochemistry, and organ morphology (Fig. 1C); network 4 includes cell death, neurological disease, and connective tissue disorders (Fig. 1D); and network 5 functions in lipid metabolism, molecular transport, and small molecule biochemistry (Fig. S1).

**Figure 1:**
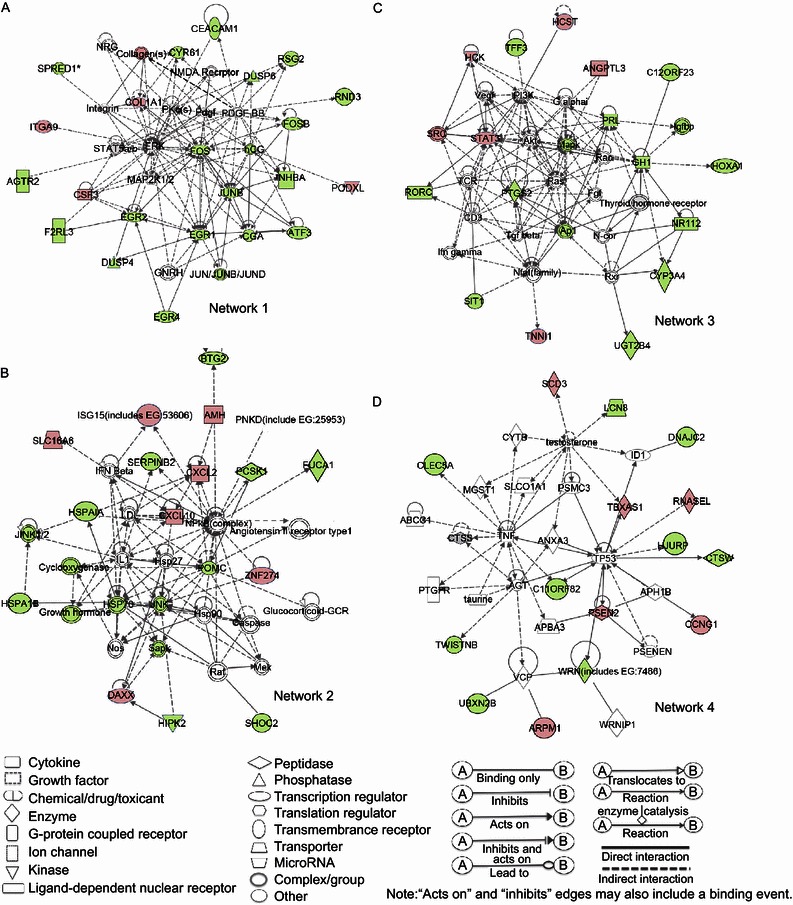
**Statistically significant signaling pathway networks between JU and vehicle groups**. Statistically significant signaling pathway networks involving the differentially expressed genes between JU and vehicle groups. (A) Network 1; (B) Network 2; (C) Network 3; (D) Network 4. The red node denotes an up-regulated gene, and the green node denotes a down-regulated gene. The intensity of colours indicates the degree of up-regulation or down-regulation

Secondly, the IPA analysis also identified 33 statistically significant canonical pathways out of 149 pathways involving the differentially expressed genes between the JU and vehicle groups (Table.S2). Of these 33 canonical pathways, the combination JU treatment regulated as many as 13 pathways independently, including renin-angiotensin signaling and cAMP-mediated signaling, whereas the JU, JA and UA groups showed 6 overlapping pathways. Meanwhile 6 overlapping pathways were identified between the JA and JU groups, and 8 overlapping pathways were discovered between the UA and JU groups (Fig. 2A). Furthermore, 10 out of 13 non-overlapping pathways regulated by JU separately were related to cerebral ischemia. These pathways were largely related to inflammation, apoptosis/necrosis, and neurogenesis/angiogenesis with regard to cerebral ischemia. However, 6 non-overlapping pathways in the JU group were stimulated by microglial activation, which plays a key role in the inflammatory reaction during cerebral ischemia (Fig. 2B). More importantly, 3 non-overlapping pathways were identified to be involved in cerebral ischemia for the first time, including molecular mechanisms of cancer, PXR/RXR activation, and caveolar-mediated endocytosis.

**Figure 2:**
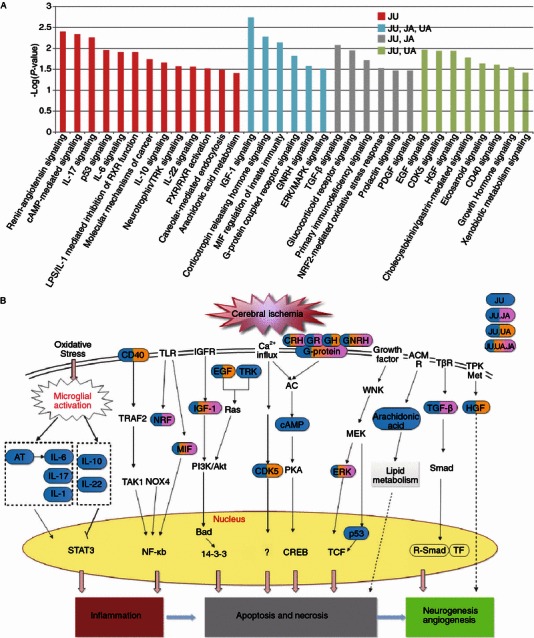
**Canonical pathways among different groups**. (A) The 33 statistically significant canonical pathways between JU and vehicle groups. The red histograms represented non-overlapping pathways in JU group. The blue histograms represented overlapping pathways among JA, UA and JU groups. The grey, green histograms represented overlapping pathways between JA and JU, UA and JU groups respectively. (B) The proposed therapeutic targets of JU group on cerebral ischemia-reperfusion injury. JU played differential roles in schematic view of known pathways. The blue ellipses within solid line represented unique canonical pathways in JU group; blue and pink ellipses within solid line represented canonical pathways in JA and JU group; blue and yellow ellipses within solid line represented canonical pathways in UA and JU group; blue, yellow and pink ellipses within solid line represented canonical pathways in JA, UA and JU group. Blue ellipses denote the potential therapeutic targets and synergistic mechanism of the combination of JA and UA

Thirdly, compared with the vehicle group, a total of 66 overlapping biological functions were identified among the JA, UA and JU groups, which accounted for 79.52 % of all the functions. Interestingly, 5 overlapping functions were discovered between the JA and UA groups, one overlapping function (amino acid metabolism) was discovered between the JA and JU groups, and 2 overlapping functions (gastrointestinal disease, renal and urological disease) were discovered between the UA and JU groups. Meanwhile, a total of 2, 4 and 3 unique functions were identified in the JA, UA and JU groups, respectively (Fig. S2). The top 10 functions of the JU group are cell death, cancer, cell cycle, cellular growth and proliferation, tissue morphology, DNA replication, recombination and repair, small molecule biochemistry, hematological disease, tissue development and hepatic system disease (Fig. S3). Moreover, according to the IPA analysis of the 66 overlapping functions among the JA, UA and JU groups, 21 functions (31.82%) belong to the category of diseases and disorders, 21 functions (31.82%) are related to molecular and cellular functions, and 24 functions (36.36%) are involved in physiological system development and function. The distribution of these functions is shown as list (Fig. S4).

In our current study, based on the analysis of the top 5 differential networks, 3 overlapping network functions were identified in the JA, UA and JU groups, including cellular compromise, small molecule biochemistry and lipid metabolism. These functions have been reported to be involved in cerebral ischemia (Simao et al., 2013; Takeda et al., 2004; Zhan et al., 2010). More importantly, we identified 6 non-overlapping network functions in the JU group, including cell cycle, drug metabolism, endocrine system development and function, organ morphology, connective tissue disorders, and molecular transport. Our results might suggest that the networks involved in the JU-dependent effects significantly vary from those of the JA and UA treatments alone in conquering cerebral ischemia.

The transformed novel activation pathways maybe involved in the synergistic mechanism of JA and UA, in which 10 pathways were related to cerebral ischemia. Oxidative stress plays a crucial role in brain damage after stroke. During cerebral ischemia-reperfusion, significant amounts of oxygen free radicals are generated, and the resting microglia in brain is activated and developed macrophage-like abilities, including phagocytosis and inflammatory responses (Ekdahl et al., 2009). Additional evidence demonstrates that microglia mediates inflammation by secreting pro-inflammatory and anti-inflammatory cytokines as well as triggering neuronal apoptosis or death in response to ischemic injury. In our study, 3 unique pathways of pro-inflammatory cytokines were identified in the JU group, including IL-6 signaling, IL-17 signaling, and the LPS/IL-1 mediated inhibition of RXR function. It was reported that the upregulated IL-6 and IL-1 may target the vascular endothelium to mediate inflammatory cascades, resulting in the aggravation of cerebral ischemic damage during the acute phase (Jafarinaveh et al., 2014). The major downstream signaling pathway of IL-6 is JAK-STAT, and STAT3 activation primarily occurs in neurons during ischemic reperfusion (Suzuki et al., 2009). STAT3 is a transcription factor that binds to DNA in the nucleus and initiates the production of more interleukins involved in the acute inflammatory response. In the experimental ischemia-reperfusion (I/R) brain model, IL-17 was verified to have pivotal roles in the evolution of brain infarction and the delayed phase of I/R injury. The induction of IL-17 was dependent on IL-23. Therefore, the activation of the 3 inflammatory signaling pathways may contribute to the synergistic mechanism of JA and UA. However, 2 unique pathways of anti-inflammatory cytokines were discovered in the JU group, including IL-10 signaling and IL-22 signaling. IL-10 and IL-22 are key members of the IL-10 cytokine family, and previous studies have highlighted the protective function of endogenous IL-10 and IL-22 during ischemic stroke through ameliorating brain inflammation and injury (Fouda et al., 2013). Additionally, IL-10 and IL-22 can directly provide protection to cortical neurons by acting on STAT3 signal transduction pathways. Traditionally, the renin-angiotensin system (RAS) has been regarded as an endocrine system that was responsible for long-term control of blood pressure, the development of hypertensive cardiomyopathy, and the development and progression of atherosclerosis. Presently, some studies have shown that RAS enhances vascular inflammation through cytokine-like actions and, through angiotensin II (AngII), induces the expression of the pro-inflammatory cytokine IL-6 (Brasier et al., 2002). Our results are the first instances of evidence that both pro-inflammatory and anti-inflammatory cytokines as well as RAS are involved in the synergistic mechanism of JA and UA. Additionally, we identified 3 novel pathways that were not originally related to cerebral ischemia, including molecular mechanisms of cancer, PXR/RXR activation, and caveolar-mediated endocytosis. Further investigations are necessary to determine the effect of these signaling pathways on the progression of cerebral ischemia.

The biological effects of JU treatment differ greatly from those of JA and UA treatment alone. Organismal survival, psychological disorders, and protein synthesis were identified as the transformed biological functions in the JU group. Previous studies demonstrated that transient cerebral ischemia triggered the suppression of protein synthesis. The phosphorylation of eukaryotic initiation factor 2α (eIF2α) and eukaryotic translation initiation factor 4GI (eIF4GI) correlates with the inhibition of protein synthesis and decreased viability at different time points during reperfusion (DeGracia et al., 2002). Either activated pERK or IREα phosphorylates eIF2α, which then inhibits most protein synthesis and may be a defense mechanism to ischemia-induced ER stress. However, the persistence of p-eIF2α coupled with eIF4G degradation may induce a pathological transition that results in a disproportion at expression of proapoptotic genes due to residual translation (Wek et al., 2006). To our knowledge, there is little evidence showing that the biochemical functions of organismal survival and psychological disorders were involved in cerebral ischemia. Further research is required to discover the correlation between the disease and these functions.

In conclusion, our integrated network analysis revealed that JA and UA treatments individually affected multiple pathways, including IGF-1 signaling. However, combining these two compounds produced a synergistic mechanism that transformed several novel activation signaling pathways, including six inflammatory pathways and cAMP signaling, as well as alteration of biological functions, such as cell cycle, endocrine system development and function, and protein synthesis.

## FOOTNOTES

This work was supported by National 11th Five-years-plan Supporting R&D Project (2006BAI08B04-06) and State Project for Essential Drug Research and Development (2013ZX09303301).

Guo Shanshan, Guo Li, Yu Yanan, Li Bing, Zhang Yingying, Li Haixia, Wu Ping, Wang Jie, Yuan Ye, Wang Zhong and Wang Yongyan declare that they have no conflict of interest. All institutional and national guidelines for the care and use of laboratory animals were followed.

## Electronic supplementary material

**Below is the link to the electronic supplementary material.**

**Supplementary material 1 (PDF 162** **kb)**
