# Supplementary material for: A partial pathway- and network-based transformation reveals the synergistic mechanism of JA and UA against cerebral ischemia-reperfusion injury
Source: Protein Cell. 2014 Sep 13;5(11):873–7. doi: 10.1007/s13238-014-0098-0 (PMC4225461; doi:10.1007/s13238-014-0098-0)
Supplement: Supplementary file 1 — Supplementary material 1 (PDF 162 kb) [file 13238_2014_98_MOESM1_ESM.pdf]

A partial pathway- and network-based transformation reveals the synergistic mechanism of JA and UA against cerebral ischemia–reperfusion injury

Guo Shanshan[1], Guo Li[2], Yu Yanan[3], Li Bing[4], Zhang Yingying[3], Li Haixia[2], Wu Ping[2], Wang Jie[2],Yuan Ye[3], Wang Zhong[3*],Wang Yongyan[3]

1 Institute of Chinese Materia Medica, China Academy of Chinese Medical Sciences, Beijing, 100700,China

2 Guang'anmen Hospital, China Academy of Chinese Medical Sciences, Beijing,100700, China

3 Institute of Basic Research in Clinical Medicine, China Academy of Chinese Medical Sciences, Beijing, 100700, China

4 Institute of Information on Traditional Chinese Medicine, China Academy of Chinese Medical Sciences, Beijing, 100700,China

Corresponding author: Prof. Zhong Wang,Institute of Basic Research in Clinical Medicine, China Academy of Chinese Medical Sciences, Dongzhimen, Beijing 100700, China,Tel: 86-10-64014411-3308, Fax: 86-10-84032881. E-Mail: zhonw@vip.sina.com

Materials and Methods

Animals

A total of 144 male Kunming mice (12 weeks old, 38–48g) were purchased from the Experimental Animal Center at the Health Science Center of Peking University and were maintained in an environmentally controlled breeding room (25°C with a 12 h light/dark cycle).Animal use protocols were reviewed and approved by the Ethics

Review Committee for Animal Experimentation, at the China Academy of Chinese Medical Sciences, and the study was carried out in accordance with the Prevention of Cruelty to Animals Act of 1986 and the guidelines from the National Institute of Health regarding the care and use of laboratory animals for experimental procedures.

MCAO model and drug treatment

Mice were subjected to the middle cerebral artery occlusion (MCAO) model, in which they were ligated with an intraluminal filament for 1.5 h and then reperfused for 24 h. The mice in the sham-operated group were surgically prepared for the insertion of the filament, but the filament was not inserted.

The animals were randomly divided into the following 6 groups: JA-treated group (50 mg/kg), UA–treated group (14 mg/kg), JU-treated group (combination of JA and UA at 1:1), NI-treated group (80 mg/kg), vehicle group (0.9% NaCl), and sham-operated group (0.9% NaCl). JA and UA were chemically standardized products from either the China Natural Institute for the Control of Pharmaceutical and Biological Products or Beijing University of Traditional Chinese Medicine, and their tested purities exceeded 98% by fingerprint chromatography. All of the drugs were dissolved in 0.9% NaCl just prior to use and injected into the tail vein of mice 1.5 h after the induction of focal cerebral ischemia. Each mouse in the vehicle and the sham-operated groups were injected with saline alone (2 ml/kg body weight) into the tail vein at the same time point.

TTC Staining and Histological analysis

The infarct volume from 9 mice from each group was measured by TTC (2, 3, 5-triphenyl tetrazolium chloride) staining. The brain was sliced into coronal sections and immersed into a 1% TTC solution for 30min at 37°C. After washing with normal saline, the slices were transferred into a 4% formaldehyde solution for fixation. Images of brain sections were captured using a digital camera (Color CCD camera TP-6001A,Topica Inc., Japan). The area of ischemic damage was measured using pathology image analysis system software (Topica Inc., Japan), and the ratio of infarct volume to the total slice was calculated.

An additional 6 mice from each group were subjected to hippocampus

histological analysis. After washing with normal saline, mouse brains were perfused with cold 4% formaldehyde for 30 min to induce polymerization. The brains were fixed in 4% formaldehyde for at least 24 h, sectioned coronally into 5-μm slices, and then stained with 0.2% thionine. The hippocampal CA1 region was observed under a microscope.

RNA isolation

The hippocampi of 9 mice from each group were homogenized in Trizol Reagent (Invitrogen, USA), and the total RNA was isolated according to the manufacturer's instructions. The purification and concentration of RNA were performed using an RNeasy Micro Kit (Qiagen, Valencia, CA) according to the manufacturer's protocol. The RNA quality was evaluated by determining the 26S/18S ratio with a bioanalyzer microchip (Agilent, Palo Alto, CA).

Microarray

Gene expression profiling was conducted using a microarray chip containing the whole genome array (Boao Capital, Beijing, China) for mice (16,463 oligoclones, Incyte Genomics, Santa Clara, CA, USA). On one given chip, each clone generated 4 technical replicates via duplicate spots printing. After smoothing spline normalization, a single intensity value for each clone was determined by averaging the quadruplet measurements. All of the clones had been verified by DNA sequencing. The total RNA from the vehicle group was labeled with Cy3 as the pool control, whereas RNA from the other groups was labeled with Cy5. The microarrays were then hybridized, washed, and scanned according to standard protocols. The experiment was repeated in triplicate, with technical quadruplets for each group within each array.

IPA analysis of Microarray Data

All microarray data were uploaded to the ArrayTrack system (Food and Drug Administration, USA).Experimental analysis was performed using the Minimum Information About a Microarray Experiment (MIAME) guidelines and the MicroArray Quality Control (MAQC) Project standards. Analysis results were submitted to the Array Express database, and the microarray data were normalized by carrying out a locally weighted linear regression (Lowess) to reduce systematic bias

as best as possible (smoothing factor: 0.2; robustness iterations: 3). One-way analysis of variance and significance analysis of the microarrays were applied to compare the means of the altered genes between the sham and vehicle, JA and vehicle, UA and vehicle, and JU and vehicle groups. Genes that showed significant changes in expression with a *P* value less than 0.05 and a fold change greater than 1.5 were selected for further analysis. Additionally, genes that had >1.5-fold increase in their expression levels were indicative of upregulation, and genes that had <0.5-fold decrease of expression levels suggested downregulation.

After the initial analysis, genes showing significant changes in expression were uploaded to the IPA system (http://www.ingenuity.com/).A cutoff was set to identify network eligible molecules whose differential expression was significantly regulated. Based on the connectivity of these network-eligible molecules, networks were algorithmically generated. The right-tailed Fisher's exact test was applied to calculate a *P* value determining if each biological function assigned to that network is due to chance alone. The significance of correlation between these genes and the canonical pathway was determined in two ways: (1) a ratio of the number of genes from the data set that map to the pathway divided by the total number of genes that map to the canonical pathway; and (2) Fisher's exact test to calculate a *P* value determining if the correlation between the genes and the canonical pathway can be explained by chance alone. A *P* value <0.05 was considered to be statistically significant. Finally, we screened out and analyzed all canonical pathways that had with a *P* value < 0.05 and a fold change >1.5.

Discussions

In our current study, based on the analysis of the top 5 differential networks, the targets genes were primarily related to ERK, NF-κB, p38 MAPK, Akt, Fos, Jnk, Ras, JUNB, EGR1, PI3K, and STAT3 in both the single and combination treatment groups. It has been reported that these genes are involved in the process of cerebral ischemia. Inhibition of the activity of p38 MAPK(mitogen-activated protein kinase)(Barone et al., 2001a; Barone et al., 2001b), NF-κB(nuclear factor-kappa B)(Nurmi et al., 2004; Xu et al., 2002), and Jnk(Murata et al.; Zhu et al.) can reduce infarct volume and

neurological deficits and improve outcome, whereas the activation of ERK(extracellular signal-regulated kinase) (Ong et al.; Sawe et al., 2008), PI3K(Brunet et al., 2001; Lu et al.), and Akt(Wu et al.; Zhao et al., 2005) provide neuroprotection in cerebral ischemia.

In recent years, the altered expression of proteins regulating the cell cycle were confirmed (resulting in cell death), and infarction was reduced significantly by inhibiting the cell cycle after cerebral ischemia(Zhang et al., 2009).Furthermore, compounds such as tricyclodecan-9-yl-xanthogenate (D609) and berberine can prevent mature neurons from entering the cell cycle at the early reperfusion, which is possibly mediated by interference due to the delayed proliferation of microglia and macrophages(Adibhatla and Hatcher; Chai et al.). Previous studies have proven that cerebral injuries can induce distinct abnormalities of the endocrine system(Schwarz et al., 2003). Dysregulation of the hypothalamic-pituitary axis can induce hypercortisolism, abolish the hormonal circadian pattern of β-endorphins and cortisol, and elevate nocturnal prolactin release(Schwab et al., 1997; Wallaschofski et al., 2006).Additionally, other abnormalities have been shown to manifest after stroke such as lowered levels of gonadotropins, growth hormone, and thyroid-stimulating hormone as well as elevated nocturnal prolactin release and impaired thyrotropin-releasing hormone-stimulated secretion of thyroid-stimulating hormone(Akhoundi et al.; Vespa). There have been few studies reporting on how the functions of drug metabolism, organ morphology and molecular transport are involved in acute cerebral ischemia. Therefore, this study may reveal novel mechanisms of the synergistic effect of JA and UA in treating cerebral ischemia.

With regard to the networks identified in the NI group(positive control), Ca^{2+} was identified as one of node molecules, which was consistent with its pharmacological effect of Ca^{2+}-antagonist against cerebral ischemia (Greenberg et al., 1990; Zhu et al., 1999). Furthermore, other node molecules, such as ERK, NF-κB, p38 MAPK, Jnk, Fos, Junb, Caspase, Ap1were also found in the NI group. It was indicated that nimodipine may act on multiple targets in treating with cerebral ischemia.

Reference

Adibhatla, R.M., and Hatcher, J.F. Protection by D609 through cell-cycle regulation after stroke. Mol Neurobiol 41, 206-217.

Akhoundi, F.H., Ghorbani, A., Soltani, A., and Meysamie, A. Favorable functional outcomes in acute ischemic stroke patients with subclinical hypothyroidism. Neurology 77, 349-354.

Barone, F.C., Irving, E.A., Ray, A.M., Lee, J.C., Kassis, S., Kumar, S., Badger, A.M., Legos, J.J., Erhardt, J.A., Ohlstein, E.H., *et al.* (2001a). Inhibition of p38 mitogen-activated protein kinase provides neuroprotection in cerebral focal ischemia. Med Res Rev 21, 129-145.

Barone, F.C., Irving, E.A., Ray, A.M., Lee, J.C., Kassis, S., Kumar, S., Badger, A.M., White, R.F., McVey, M.J., Legos, J.J., *et al.* (2001b). SB 239063, a second-generation p38 mitogen-activated protein kinase inhibitor, reduces brain injury and neurological deficits in cerebral focal ischemia. J Pharmacol Exp Ther 296, 312-321.

Brunet, A., Datta, S.R., and Greenberg, M.E. (2001). Transcription-dependent and -independent control of neuronal survival by the PI3K-Akt signaling pathway. Curr Opin Neurobiol 11, 297-305.

Chai, Y.S., Hu, J., Lei, F., Wang, Y.G., Yuan, Z.Y., Lu, X., Wang, X.P., Du, F., Zhang, D., Xing, D.M., *et al.* Effect of berberine on cell cycle arrest and cell survival during cerebral ischemia and reperfusion and correlations with p53/cyclin D1 and PI3K/Akt. Eur J Pharmacol 708, 44-55.

Greenberg, J.H., Uematsu, D., Araki, N., Hickey, W.F., and Reivich, M. (1990). Cytosolic free calcium during focal cerebral ischemia and the effects of nimodipine on calcium and histologic damage. Stroke 21, IV72-77.

Lu, C., Liu, L., Chen, Y., Ha, T., Kelley, J., Schweitzer, J., Kalbfleisch, J.H., Kao, R.L., Williams, D.L., and Li, C. TLR2 ligand induces protection against cerebral ischemia/reperfusion injury via activation of phosphoinositide 3-kinase/Akt signaling. J Immunol 187, 1458-1466.

Murata, Y., Fujiwara, N., Seo, J.H., Yan, F., Liu, X., Terasaki, Y., Luo, Y., Arai, K., Ji, X., and Lo, E.H. Delayed inhibition of c-Jun N-terminal kinase worsens outcomes after focal cerebral ischemia. J Neurosci 32, 8112-8115.

Nurmi, A., Lindsberg, P.J., Koistinaho, M., Zhang, W., Juettler, E., Karjalainen-Lindsberg, M.L., Weih, F., Frank, N., Schwaninger, M., and Koistinaho, J. (2004). Nuclear factor-kappaB contributes to infarction after permanent focal ischemia. Stroke 35, 987-991.

Ong, W.Y., Ng, M.P., Loke, S.Y., Jin, S., Wu, Y.J., Tanaka, K., and Wong, P.T. Comprehensive gene expression profiling reveals synergistic functional networks in cerebral vessels after hypertension or hypercholesterolemia. PLoS One 8, e68335.

Sawe, N., Steinberg, G., and Zhao, H. (2008). Dual roles of the MAPK/ERK1/2 cell signaling pathway after stroke. J Neurosci Res 86, 1659-1669.

Schwab, S., Spranger, M., Krempien, S., Hacke, W., and Bettendorf, M. (1997). Plasma insulin-like growth factor I and IGF binding protein 3 levels in patients with acute cerebral ischemic injury. Stroke 28, 1744-1748.

Schwarz, S., Schwab, S., Klinga, K., Maser-Gluth, C., and Bettendorf, M. (2003). Neuroendocrine changes in patients with acute space occupying ischaemic stroke. J Neurol

Neurosurg Psychiatry 74, 725-727.
Vespa, P.M. Hormonal dysfunction in neurocritical patients. Curr Opin Crit Care 19, 107-112.
Wallaschofski, H., Lohmann, T., Hild, E., Kobsar, A., Siegemund, A., Spilcke-Liss, E., Hentschel, B., Stumpf, C., Daniel, W.G., Garlichs, C.D., *et al.* (2006). Enhanced platelet activation by prolactin in patients with ischemic stroke. Thromb Haemost 96, 38-44.
Wu, J., Li, Q., Wang, X., Yu, S., Li, L., Wu, X., Chen, Y., Zhao, J., and Zhao, Y. Neuroprotection by curcumin in ischemic brain injury involves the Akt/Nrf2 pathway. PLoS One 8, e59843.
Xu, L., Zhan, Y., Wang, Y., Feuerstein, G.Z., and Wang, X. (2002). Recombinant adenoviral expression of dominant negative IkappaBalpha protects brain from cerebral ischemic injury. Biochem Biophys Res Commun 299, 14-17.
Zhang, Q., Chen, C., Lu, J., Xie, M., Pan, D., Luo, X., Yu, Z., Dong, Q., and Wang, W. (2009). Cell cycle inhibition attenuates microglial proliferation and production of IL-1beta, MIP-1alpha, and NO after focal cerebral ischemia in the rat. Glia 57, 908-920.
Zhao, H., Shimohata, T., Wang, J.Q., Sun, G., Schaal, D.W., Sapolsky, R.M., and Steinberg, G.K. (2005). Akt contributes to neuroprotection by hypothermia against cerebral ischemia in rats. J Neurosci 25, 9794-9806.
Zhu, D., Li, R., Liu, G., and Hua, W. (1999). Nimodipine inhibits calcium-independent nitric oxide synthase activity in transient focal cerebral ischemia rats and cultured mouse astroglial cells. Life Sci 65, PL221-231.
Zhu, J., Shen, W., Gao, L., Gu, H., Shen, S., Wang, Y., Wu, H., and Guo, J. PI3K/Akt-independent negative regulation of JNK signaling by MKP-7 after cerebral ischemia in rat hippocampus. BMC Neurosci 14, 1.

Supplementary Figure S1 Statistically significant signaling pathway networks 5 between JU and vehicle groups. The red node denotes an up-regulated gene, and the green node denotes a down-regulated gene. The intensity of colours indicates the degree of up-regulation or down-regulation.

Supplementary Figure S2 The Wayne figures of overlapping and different biological functions among JA, UA and JU groups. JA (red round) denoted JA vs.vehicel groups, UA (yellow round) denotes UA vs. vehicle groups, JU (blue round) denotes JU vs. vehicle groups. Only significant functions are shown in this figure based on a *P* value <0.05 and a fold change >1.5 between groups.

Supplementary Figure S3 Top 10 significant biological functions between JU and vehicle groups.The yellow vertical line indicates a threshold of P<0.05.

Supplementary Figure S4 The pie charts of distributions of the 66 overlapping functions among JA,UA and JU groups.

Supplementary Figure 5 Statistically significant signaling pathway networks between NI and vehicle groups. Statistically significant signaling pathway networks involving the differentially expressed genes between NI and vehicle groups, with functions in (A) molecular transport, small molecule biochemistry, DNA replication, recombination, and repair (Network 1); (B) endocrine system disorders, gene expression, hematological disease (Network 2); (C) DNA replication, recombination, and repair, genetic disorder, metabolic disease (Network 3); (D)gene expression, cancer, hepatic system disease (Network 4); and (E) cell signaling, molecular transport, vitamin and mineral metabolism (Network 5). The red node denotes an up-regulated gene, and the green node denotes a down-regulated gene. The intensity of colours indicates the degree of up-regulation or down-regulation.

Supplementary Table 1 Top 5 differential networks between JU and vehicle groups.

Supplementary Table 2 Differential canonical pathways between JU and vehicle groups.
